# Cytotoxicity of Metal‐Based Nanoparticles: From Mechanisms and Methods of Evaluation to Pathological Manifestations

**DOI:** 10.1002/advs.202106049

**Published:** 2022-03-27

**Authors:** Peizheng Xiong, Xiangming Huang, Naijing Ye, Qunwen Lu, Gang Zhang, Shunlin Peng, Hongbo Wang, Yiyao Liu

**Affiliations:** ^1^ TCM Regulating Metabolic Diseases Key Laboratory of Sichuan Province Hospital of Chengdu University of Traditional Chinese Medicine Chengdu 610072 P. R. China; ^2^ The First Affiliated Hospital of Guangxi University of Traditional Chinese Medicine Nanning Guangxi Province 530023 P. R. China; ^3^ Institute of Smart City and Intelligent Transportation Southwest Jiaotong University Chengdu 611700 P. R. China; ^4^ State Key Laboratory of Electronic Thin Film and Integrated Devices University of Electronic Science and Technology of China Chengdu 610054 P. R. China; ^5^ Department of Biophysics School of Life Science and Technology University of Electronic Science and Technology of China Chengdu Sichuan 610054 P. R. China

**Keywords:** cytotoxic effect, cytotoxicity, evaluation method, metal‐based nanoparticle, toxic mechanism

## Abstract

Metal‐based nanoparticles (NPs) are particularly important tools in tissue engineering‐, drug carrier‐, interventional therapy‐, and biobased technologies. However, their complex and varied migration and transformation pathways, as well as their continuous accumulation in closed biological systems, cause various unpredictable toxic effects that threaten human and ecosystem health. Considerable experimental and theoretical efforts have been made toward understanding these cytotoxic effects, though more research on metal‐based NPs integrated with clinical medicine is required. This review summarizes the mechanisms and evaluation methods of cytotoxicity and provides an in‐depth analysis of the typical effects generated in the nervous, immune, reproductive, and genetic systems. In addition, the challenges and opportunities are discussed to enhance future investigations on safer metal‐based NPs for practical commercial adoption.

## Graphical abstract

Considerable experimental and theoretical efforts have been directed toward understanding the cytotoxicity of metal‐based nanoparticles but additional efforts are still needed before metal‐based nanoparticles can be routinely used in clinical medicine field. This review summarizes the state of toxic mechanisms and evaluation methods of cytotoxicity, and gives an in‐depth analysis of typical cytotoxic effects.

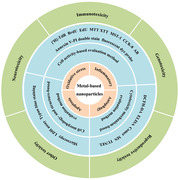

## 1 Introduction

Over the recent decades, nanotechnology has been integrated in many areas of research and industry, including nanomedicine (e.g., antibacterial, bioimaging, and photothermal therapy),^[^ ^1^, ^2^, ^3^ ^]^ industrial chemistry (e.g., catalysis, energy conversion, and sensors), and the production of basic consumer goods (e.g., cosmetics, sunscreens, and textiles). Nanomaterials have become increasingly popular given that they have improved the performance and functionality of several technologies. However, much remains unknown of the short‐ and long‐term effects of nanomaterials on human and ecosystem health. Compared to ordinary materials, nanomaterials exhibit unique physical and chemical properties that cause different biochemical activities and are associated with distinct cytotoxic effects and mechanisms. Nanomaterials are far smaller than cells, which allows them to easily penetrate cell membranes and interact with cells or subcellular organelle structures, ultimately resulting in neurotoxicity, immunotoxicity, and genotoxicity, among others. Their widespread use increases the risk and pathways for exposure (e.g., inhalation, oral ingestion, and skin contact). Uncertainties regarding the safe use of nanomaterials are considered a major obstacle to innovation and investment in clinical medicine.^[^ ^4^ ^]^ Given that most purified carbon nanomaterials are nontoxic in many cell lines, this review focused on the cytotoxicity of metal‐based nanoparticles (NPs).

Considerable experimental and theoretical efforts have been directed toward understanding the cytotoxic effects and interaction mechanisms of metal‐based NPs; however, the potential risks associated with metal‐based NPs do not allow their routine use in clinical medicine. Metal‐based NPs exhibit size, surface, and quantum properties that may lead to abnormal features in adsorption,^[^ ^5^ ^]^ chemical reaction,^[^ ^6^ ^]^ dispersion or agglomeration,^[^ ^7^, ^8^ ^]^ and tissue penetration.^[^ ^9^ ^]^ The cytotoxicity of metal‐based NPs depends on their composition, size, shape, surface charge, solubility, coating material, and reactivity.^[^ ^10^ ^]^ Even metal‐based NPs with the same chemical composition may show significant differences in their toxicological properties,^[^ ^11^, ^12^ ^]^ which complicate quantitative risk assessments for their use in clinical settings. In this regard, a systematic review of the cytotoxicity of metal‐based NPs can provide guidance in the development, functionalization, application, and safety evaluation of metal‐based NPs.

**Figure 1:**
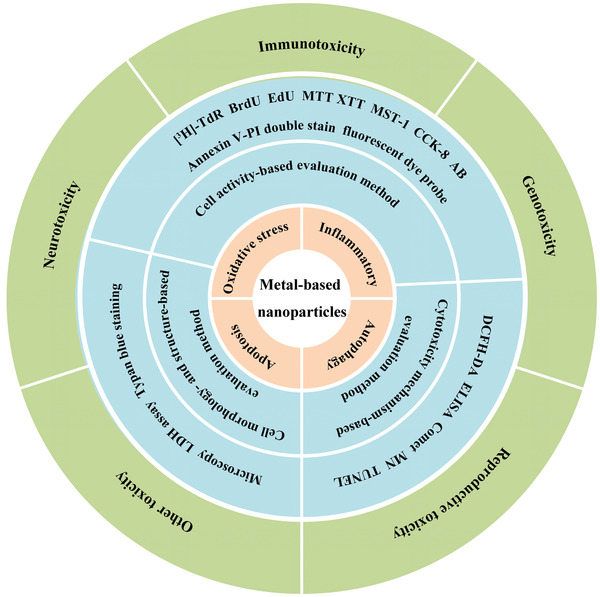
The cytotoxicity induced by metal‐based NPs with regards to mechanisms (light brown), evaluation methods (blue), and pathological manifestations (green).

Previous reviews have focused on the cytotoxicity of a certain metal‐based NP or the cytotoxic effects observed in specific tissues and lack any thorough assessment of systematic toxicity evaluation methods, toxicity mechanisms based on signal pathways, or pathological manifestations in multiple organs. To provide a more comprehensive systematic review, we first summarize the key signaling pathways of various cytotoxic mechanisms and then present the principles, advantages, and limitations of different cytotoxic evaluation methods. Furthermore, we describe the corresponding cytotoxic pathological manifestations of metal‐based NPs (**Figure** **1**). Finally, to elucidate the potential risks of metal‐based NPs, we discuss the current challenges in cytotoxicity research and provide some perspectives on the safety evaluation of metal‐based NPs.

## 2 Cytotoxicity Mechanism of Metal‐Based NPs

**Figure 2:**
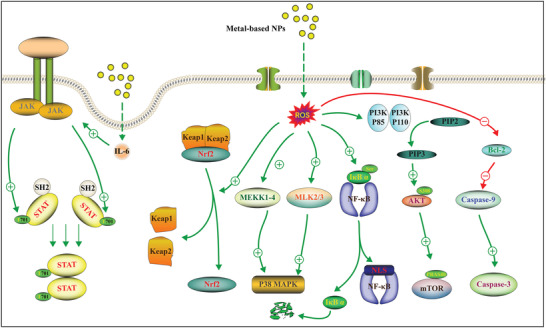
Typical mechanism of cytotoxicity induced by metal‐based NPs.

Metal‐based NPs usually produce unique biological effects at the cellular level by affecting transmembrane and basic cellular processes, such as cell division, proliferation, apoptosis, and regulation of signal transduction pathways.^[^ ^13^, ^14^ ^]^ So far, no definite conclusions have been reported on the cytotoxicity mechanism induced by metal‐based NPs. Oxidative stress and inflammation are the main explanatory factors in cytotoxicity, which have attracted considerable attention. Oxidative stress is characterized by an excessive production of reactive oxygen species (ROS), which alters the oxidation‐reduction state, while inflammation is a protective response to infection, damage, or stress, but can also have harmful effects if unregulated.^[^ ^15^, ^16^ ^]^ In recent years, autophagy and apoptosis have been extensively studied as mechanisms of cytotoxicity in metal‐based NPs. Although cytotoxic mechanisms differ, they are still fundamentally related. For example, ROS can activate and recruit inflammatory cells, promote inflammatory responses, which in turn can exacerbate ROS production.^[^ ^17^ ^]^ In turn, excessive ROS can induce mitochondrial dysfunction, oxidative damage, and apoptosis and disturb the balance of the redox system.^[^ ^18^ ^]^ In addition, cytotoxicity mediated by metal‐based NPs involves the synergy of multiple signaling pathways. For example, ZnO NPs can induce neurotoxicity by activating the transcription factor nuclear factor‐kappa B (NF‐*κ*B), extracellular signal‐regulated kinase (ERK), and p38 mitogen activated protein kinases (MAPK) signaling pathways,^[^ ^19^ ^]^ while the immunotoxicity induced by Cu NPs is related to the activation of nuclear factor erythroid 2‐related factor 2 (Nrf2), MAPK, and phosphoinositide 3‐kinase (PI3K)/protein kinase B (AKT).^[^ ^20^ ^]^ In this section, we summarize the main toxic signaling pathways of metal‐based NPs and discuss their relationship with oxidative stress, inflammatory response, autophagy, and apoptosis (**Figure** **2**).

### 2.1 Oxidative Stress

Metal‐based NPs are prone to oxidation‐reduction reactions because of their unique physical and chemical properties, such as small particle size, large specific surface area, and strong surface activity, which generate free radicals and cause oxidative stress.^[^ ^21^, ^22^ ^]^ There is increasing evidence that the intracellular regulation of antioxidant levels is closely related to ROS level and oxidant source.^[^ ^23^ ^]^ ROS is an important physiological regulator of intracellular signaling pathways. However, high levels of ROS can disrupt the balance of oxidation and antioxidant systems and generate cellular oxidative stress, thereby causing lipid peroxidation, protein and DNA damage, and signaling pathway disorders.^[^ ^23^ ^]^ Under high ROS content, Nrf2 and P38 MAPK signaling pathway are commonly involved in the generation of antioxidants.^[^ ^24^, ^25^ ^]^

#### 2.1.1 Nrf2/ARE Signaling Pathway

The Nrf2/ARE signaling pathway is an important frontal endogenous defense system of the human body and plays a protective role against oxidative stress. ARE is a DNA promoter‐binding sequence located in the upstream regulatory region of some protective genes, whereas Nrf2 is the activator of this sequence. Under normal physiological conditions, Nrf2 binds to its inhibitor, Kelch‐like ECH‐associated protein 1 (Keap1), and exists in the cytoplasm in an inactive state. During oxidative stress, Nrf2 uncouples from Keap1 and translocates into the nucleus where it induces the transcription of a series of cytoprotective genes and activates the defense system. Thus, the Keap1‐Nrf2‐ARE signaling pathway regulates the redox state of cells to maintain cell homeostasis.^[^ ^26^ ^]^

In this signaling pathway, Nrf2 is a key transcription factor involved in oxidative stress‐induced damage. A variety of metal‐based NPs (e.g., Au, TiO_2_, and Ag) can increase ROS and malondialdehyde (MDA) levels, which stimulates the expression of Nrf2 signal transduction pathways and downstream genes.^[^ ^27^, ^28^, ^29^ ^]^ Heme oxygenase‐1 (HO‐1) is the most widely upregulated antioxidant gene that is activated by Nrf2. Its deletion reduced the cytoprotective effect of Nrf2 in endothelial cells treated with ZnO NPs, indicating that the Nrf2‐HO‐1 axis is involved in ZnO NP‐induced endothelial injury. The key regulatory mechanism of the antioxidant response also showed that ROS production induced by ZnO NPs can increase Nrf2 in a dose‐ and time‐dependent manner.^[^ ^30^ ^]^ Glutathione (GSH), another antioxidant, responds in a similar manner to Nrf2 protein expression in conditions of oxidative stress. Following exposure to Fe‐Si NPs at 25 and 50 µg mL^‐1^ for 24 and 72 h, Nrf2 protein expression in Caco2 cells increased with the ROS and GSH levels.^[^ ^31^ ^]^ The antioxidant Nrf2 pathway may not be sufficient in ameliorating the oxidative damage induced by metal‐based NPs. In particular, long‐term exposure to high‐doses of metal NPs downregulated Nrf2 and HO‐1 gene expression in human subjects.^[^ ^32^, ^33^, ^34^ ^]^

#### 2.1.2 MAP Kinases‐Based Signaling Pathway

MAPKs consist of three main subgroups: p38, c‐Jun N‐terminal kinase (JNK), and ERK. MAPK activation is mediated by a three‐layer kinase module through continuous protein phosphorylation, which includes MAPK kinase kinases (MAP3Ks), MAPK kinases (MAP2Ks), and MAPK.^[^ ^35^ ^]^ Notably, ROS could activate MAPK, and the inhibition of p38 MAPK reduced ROS‐induced cytotoxicity, thus regulating oxidative stress, gene transcription, and immune response.^[^ ^36^, ^37^ ^]^

The signaling pathways through which p38 activates oxidative stress have been clearly defined in mammalian systems. Current evidence suggests that metal‐based NPs can induce oxidative stress through p38. For example, Ti‐based NPs are widely used in various fields due to their excellent properties.^[^ ^38^, ^39^ ^]^ After exposure to TiO_2_ NPs, the expression levels of p38 and JNK were increased, indicating that TiO_2_ NP‐induced oxidative stress activated the Nrf2, p38, and JNK MAPK signaling pathways.^[^ ^40^ ^]^ Similarly, when Ag NPs are used as stressors, *Caenorhabditis elegans* could regulate the oxidative stress response through the p38 MAPK pathway.^[^ ^41^ ^]^

Nicotinamide adenine dinucleotide phosphate oxidase (NOX) and ERK are closely related to ROS generation and the MAPK signaling pathway and exhibit certain changes under oxidative stress induced by metal‐based NPs. For example, Au NPs can increase the production of ROS in a p38‐ and ERK‐dependent manner by upregulating and activating NOX2.^[^ ^42^ ^]^ In addition, metal oxides (such as ZnO NWs) have large bandgaps and are widely used in ultraviolet photodetectors.^[^ ^43^ ^]^ However, researchers found that long‐term exposure to ultraviolet radiation B (UVB) and ZnO NPs can lead to continuous ROS generation, thereby causing oxidative stress and DNA damage through the MAPK signaling pathway, which is associated with the enhanced dermal penetration of ZnO NPs under UVB exposure.^[^ ^44^ ^]^

### 2.2 Inflammatory Response

Metal‐based NPs may cause the release of a variety of inflammatory factors, such as IL‐1, IL‐6, and TNF‐*α*. During this inflammatory response, cytokines and chemokines determine the degree of the inflammation cascade. Hence, describing the degree of the inflammatory response mediated by metal‐based NPs is important for the development of safe NPs. Here, we review the main signaling pathways involved in the metal‐based NP‐induced inflammatory response to elucidate the underlying mechanism.

#### 2.2.1 NF‐*κ*B Signaling Pathway

NF‐*κ*B is a key factor in the regulation of gene transcription. It plays an important role in immunity and inflammation and regulates more than 100 proinflammatory genes.^[^ ^45^ ^]^ NF‐*κ*B includes five members that generate dimeric transcription factors: NF‐*κ*B2 p52/p100, NF‐*κ*B1 p50/plo5, c‐Rel, RelA/p65, and RelB. In the classical pathway, the dimeric transcription factor NF‐*κ*B/Rel binds to and is simultaneously inactivated by I*κ*B in the cytoplasm. When cells receive external stimuli, the I*κ*B protein is ubiquitinated, the lysosome is degraded, and NF‐*κ*B/Rel is released. Subsequently, the activated NF‐*κ*B/Rel complex enters the nucleus to induce gene expression following phosphorylation and modification.

Myocardial cell swelling and inflammatory cell infiltration have been observed in murine heart tissue exposed to TiO_2_ NPs, where a significant increase in NF‐*κ*B promoted the expression of IL‐1*β* and TNF‐*α*.^[^ ^46^ ^]^ Blocking of NF‐*κ*B gene expression could lead to the downregulation of IL‐6 and TNF‐*α*, indicating that the inflammatory response is mediated by the NF‐*κ*B pathway.^[^ ^47^ ^]^ Furthermore, TiO_2_ NPs triggered NF‐*κ*B activation and subsequently induced the expression of inflammatory cytokines by stimulating Ca^2+^/PKC/p38 MAPK cascade reactions.^[^ ^48^ ^]^

The activation of NF‐*κ*B depends on the phosphorylation of I*κ*B kinase (IKK), which contains two main kinase subunits, IKK*α* and IKK*β*. Metal‐based NPs, such as Ag NPs, significantly increased the phosphorylation of IKK*α*/*β* and I*κ*B*α*, leading to the release and nuclear translocation of NF‐*κ*B.^[^ ^49^ ^]^ In a study of nerve inflammation caused by ZnO NPs, it was found that NF‐*κ*B activation was induced by a Ca^2+^‐dependent pathway.^[^ ^19^ ^]^ Furthermore, by comparing the ability of CdTe quantum dots (QDs), Ag, and Au NPs to stimulate NF‐*κ*B binding in HepG2 cells, researchers found that the Au NPs and CdTe QDs strongly inhibited NF‐*κ*B binding activity. Co‐treatment with Ag NPs and CdTe QDs resulted in an additive effect, whereas the presence of Ag NPs weakened the inhibitory effect of Au NPs.^[^ ^50^ ^]^ Notably, grape seed proanthocyanidin extract and *Lactobacillus rhamnosus* effectively inhibited the NF‐*κ*B signaling pathway.^[^ ^51^, ^52^, ^53^ ^]^

#### 2.2.2 JAK/STAT Signaling Pathway

The Janus kinase (JAK) and signal transducer and activator of transcription (STAT) pathways play key roles in the regulation of cell growth, differentiation, survival, and pathogen resistance.^[^ ^54^ ^]^ The JAK/STAT pathway comprises three components: signal‐receiving tyrosine kinase‐related receptor, signal‐transmitting tyrosine kinase JAK, and transcription factor STAT.^[^ ^55^ ^]^ This pathway is the main signal transduction mechanism for a variety of cytokines and growth factors. As previously stated, inflammation is a natural response to tissue and cell damage caused by pathogens, noxious stimuli, and physical injury.^[^ ^56^ ^]^ It involves a variety of inflammatory cytokines, including IL‐6, IL‐8, IL‐10, and TNF‐*α*, that induce a series of cellular responses.

STAT3 is a transcription factor that plays an important role in inflammatory responses. Binding of IL‐6 to its homologous cell surface receptor (IL‐6R) stimulates the phosphorylation of STAT3, which stimulates IL‐6 production. A study has found that exposure to Ni NPs produces high levels of acute proinflammatory cytokines, while the susceptibility of male mice to acute neutrophil inflammation was related to the induction of IL‐6 and CXCL1 and an increase in STAT3 activation.^[^ ^57^ ^]^ Ag NP hydrogels can upregulate interferon‐inducible protein (IFI), IFI with tetratricopeptide repeats (IFIT), and interleukin (IL) in the tetratricopeptide (IFIT) family. Although these inflammatory factors participate as ligands in the immune response pathway, they may also activate the JAK‐STAT signaling pathway by binding to JAK receptors.^[^ ^58^ ^]^ The myocarditis induced by TiO_2_ NPs may be related to a variety of STAT protein families, including STAT1, STAT3, and STAT6.^[^ ^46^ ^]^ In addition, TiO_2_ NPs activated JAK2 kinase and phosphorylated the STAT3 protein, thereby inducing the expression of IL‐6.^[^ ^59^ ^]^ On the other hand, PTPN6 is a key negative regulator of intracellular signal transduction and its inhibition can increase oxidative stress and exacerbate chronic inflammatory airway disease. In Al_2_O_3_ NP exposure, the inhibition of PTPN6 and phosphorylation of STAT3 exacerbated lung inflammation.^[^ ^60^ ^]^ Hence, PTPN6 is considered to be a negative regulator of STAT3 in multiple cell lines.

### 2.3 mTOR Signaling Pathway Mediated Autophagy

Autophagy is a lysosomal degradation pathway that determines cell survival, differentiation, and development and is essential for homeostasis. Autophagy plays an adaptive role in protecting organisms from various pathologies, including infection, cancer, heart disease, neurodegeneration, and aging. However, excessive autophagy can cause cell damage or death.^[^ ^61^ ^]^ In mammals, the rapamycin (mTOR) pathway is the main signaling pathway of autophagy, since the formation and maturation of autophagy depend on mTOR, and its maladjustment is necessary for autophagy.^[^ ^62^ ^]^ mTOR includes mTORC1 and mTORC2. mTORC1 promotes cell growth and metabolism and inhibits autophagy by binding to unc‐51‐like kinase 1 (ULK1). mTORC2 inhibits autophagy via the serine/threonine protein kinase sgk1 protein and promotes autophagy by upregulating the expression of mitochondrial voltage‐dependent anion‐selective channel protein 1. The PI3K/AKT pathway is the most common upstream pathway of mTOR during autophagy. Activated PI3K and mTORC2 can activate AKT, which triggers the downstream mTOR to regulate autophagy. The autophagy toxicities of various NPs have been extensively studied and their potential mechanisms of action proposed. Ultrastructural studies have shown that Ag NPs could induce autophagosome accumulation and vacuolization in a mouse hippocampal neuronal cell line (HT22). An in‐depth mechanistic study indicated that the phosphorylation of PI3K and AKT could be enhanced by exposure to Ag NPs, thus increasing the phosphorylation of mTOR and decreasing its expression. The neurotoxicity induced by Ag NPs may be related to the activation of the PI3K/AKT/mTOR signaling pathway and the induction of autophagy by ROS production in HT22 cells.^[^ ^63^ ^]^

AMPK is another important upstream protein of mTOR that plays a negative regulatory role in mTOR activation. Sublethal exposure to Ag NPs can inhibit mTOR signal transduction by activating the AMPK/ACC1 pathway. The AMPK/mTOR signaling pathway was also involved in the activation of autophagy induced by Ag NPs at low concentrations.^[^ ^64^ ^]^ Notably, numerous studies have proposed that autophagy induced by Ag NPs simultaneously induced cell death and protected cells from the toxicity of NPs. This phenomenon may be related to the removal of protein aggregates and damaged organelles through autophagy,^[^ ^65^ ^]^ though additional research into the effect of Ag NP‐induced autophagy on cell death is required. Autophagy induced by ZnO NPs can promote the delivery of ZnO NPs to lysosomes, while the acidic environment of the lysosome promoted the dissolution of ZnO NPs and the sequential release of Zn ions, thus, directly inducing cell death.^[^ ^66^ ^]^ Similarly, PI3K/AKT and MAPK have been associated with ZnO NP‐induced autophagy through various cellular stress mechanisms.^[^ ^67^ ^]^ Some studies suggest that autophagy induced by other metal‐based NPs, such as TiO_2_ and Au NPs, may also be related to the mTOR signaling pathway.^[^ ^68^, ^69^ ^]^

### 2.4 Caspase Signaling Pathway‐Mediated Apoptosis

Apoptosis is a form of gene‐regulated cell death that plays a role in several biological processes, including embryogenesis, aging, and pathogenesis. It participates in the molecular mechanisms of death signals, gene regulation, and effector activation.^[^ ^70^ ^]^ Two main apoptotic pathways have been proposed: the extrinsic or death receptor pathway, and the intrinsic or mitochondrial pathway. In addition, the two pathways are directly linked, with molecules in one pathway being able to influence the other. Both the extrinsic and intrinsic apoptotic pathways exert their effects through the action of a serine protease caspase.^[^ ^71^ ^]^ Normally, caspases exist as inactive zymogens (procaspases) and are activated by an apoptotic stimulus.

Caspase‐3 is regarded as an apoptosis “effector,” since it is involved in the initiation of the “death cascade,” and can be upregulated by exposure to metal‐based NPs. For instance, increased levels of caspase‐3 expression were observed in mouse brain regions exposed to Fe_2_O_3_ NPs, which confirmed apoptotic activity.^[^ ^72^ ^]^ Similarly, the apoptosis of human retinal cells has been related to the endogenous pathway of caspase‐3 after exposure to Ag NPs in a dose‐dependent manner.^[^ ^73^ ^]^ In addition, the activity of caspase‐3 correlated positively with the size of Ag NPs.^[^ ^74^ ^]^ Caspase‐9 is an initial caspase that recruits and activates caspase‐3, thereby initiating the caspase cascade reaction and inducing the apoptosis of target cells. Several recent reports have shown that apoptosis induced by ZnO NP exposure was closely related to the activation of caspase‐3, caspase‐7, and caspase‐9.^[^ ^75^, ^76^ ^]^ TiO_2_ NPs also induced cell apoptosis through nuclear pyknosis, activation of caspase‐3, upregulation of the proapoptotic Bax protein, and downregulation of the anti‐apoptotic Bcl‐2 protein.^[^ ^77^, ^78^ ^]^ The size of TiO_2_ NPs played an important role in determining their cytotoxicity, and larger NPs usually induced stronger apoptosis.^[^ ^79^ ^]^ Alarifi et al. also observed that the expression of both mRNA and protein levels of Bax were upregulated, while the expression of bcl‐2 was downregulated in human neuronal cells treated with gadolinium oxide NPs.^[^ ^80^ ^]^ The apoptosis induced by other metal‐based NPs, such as Au, CdS, and NiO NPs, has also been related to the caspase signaling pathway.^[^ ^81^, ^82^, ^83^ ^]^

## 3 Methods for Evaluating the Cytotoxicity of Metal‐Based NPs

NPs are widely used in high‐performance energy storage and conversion, catalysis, and other chemical applications, which undoubtedly increases the risk of exposure and biological safety issues.^[^ ^84^, ^85^ ^]^ Therefore, innovative methods to evaluate the biological safety of NPs are urgently needed. Owing to significant differences in the physicochemical characteristics as well as a lack of standards in experimental doses and conditions, no standard measurement technique exists for the evaluation of metal‐based NP‐induced cytotoxicity. Therefore, careful consideration of feasibility, sensitivity, and limitations should be made when selecting a method for evaluating NP content. Here, we systematically summarize the advantages and limitations of commonly used detection methods according to cell morphology, structure, activity, and toxicity mechanism.

### 3.1 Cell Morphology‐ and Structure‐Based Evaluation Method

**Figure 3:**
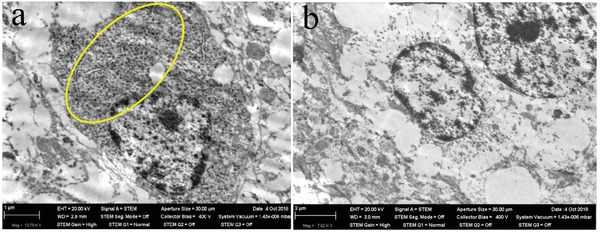
STEM images of a neuron in a rat olfactory bulb after 3 months of inhalation exposure to NiO NPs at a concentration of 0.23 mg m^‐3^. a) NiO NPs retention. b) Ultrastructural autolysis. Reproduced with permission.^[^ ^87^ ^]^ Copyright 2019, Molecular Diversity Preservation International (MDPI).

The effect of metal‐based NPs on cell morphology and structure can be determined using several distinct techniques (**Table** **1**). Cell morphology can be observed by microscopy to determine cell folding, rounding, or destruction (**Figure** **3**). The destruction of the cell membrane releases enzymes, including lactate dehydrogenase (LDH), into the cytoplasm.^[^ ^86^ ^]^ Hence, quantitative analysis of cytotoxicity can be achieved by detecting the activity of LDH released into a culture medium from plasma membrane‐ruptured cells. LDH release is regarded as an important indicator of cell membrane integrity and has been widely used for cytotoxicity evaluation. In addition, the integrity of cell membranes can be determined by measuring the uptake of exogenous substances by living cells. For example, normal cells are not stained by trypan blue because the particles cannot pass through cell membranes, whereas dead cells are easily stained.

**Table 1:** Typical characteristics of evaluation methods for cell morphology and structures

Index	Method	Advantages	Limitations	Refs.
Cell morphology	Microscopy	Simple, intuitive, low cost	Subjective, low accuracy	[88]
cell membrane integrity	LDH assay	Simple, sensitive	Sensitive to environment (e.g., temperature and pH)	[89]
	Trypan blue staining	Rapid, low cost	Potentially carcinogenic	[90]

### 3.2 Cell Activity‐Based Evaluation Method

Cell activity is an important indicator of normal cell growth under certain conditions. In this section, we summarize the most commonly used indexes and methods for evaluating cell activity based on cell growth, metabolism, and apoptosis. Many advanced cell activity‐based methods have been developed to evaluate cytotoxicity, including 3‐(4,5‐dimethylthiazol‐2‐yl)‐2,5‐diphenyltetrazolium bromide (MTT), 2,3‐bis‐(2‐methoxy‐4‐nitro‐5‐sulphenyl)‐(2H)‐tetrazolium‐5‐carboxanilide (XTT), and alamar blue (AB).^[^ ^91^, ^92^, ^93^ ^]^ The MTT method is simple, fast, and accurate but is not suitable for suspended cells. The XTT method is relatively more expensive, although the reaction product is water soluble and it does not require the use of a pyrolysis solution to dissolve the precipitate. AB is safe and nontoxic, but requires more training and is, therefore, less user friendly. Below, we provide a systematic summary of these methods (**Table** **2**).

**Table 2:** Characteristics of typical evaluation methods for cell activity

Index	Method	Advantages	Limitations	Refs.
DNA synthesis rate	[^3^H]‐TdR	Objective, reproducible, accurate	Expensive, radioactive, contamination risk, cell damage	[118]
	BrdU	High accuracy	Destroys the DNA double‐strand structure	[119]
	EdU	Rapid, no DNA denaturation, no additional protease treatment	Requires fluorescent microscopy or flow cytometry	[120]
Metabolic activity	MTT	High efficiency, simple, suitable for mass detection	Not applicable to suspended cells	[91]
	XTT MST‐1 CCK‐8	Reaction product is water‐soluble, suitable for cell suspensions, high efficiency	Expensive, Instability of the XTT aqueous solution	[121, 122, 123]
	AB	High specificity and sensitivity, repeatability	Requires training, expensive	[124]
Cell apoptosis	Annexin V‐PI double stain	Effective, rapid, easy to use	Expensive reagents	[123]
MMP	Fluorescent dye probe	Sensitivity of Rhod 123 and JC‐1	Not suitable for in situ analysis	[125]

#### 3.2.1 Evaluation Methods for Cell Growth Ability

DNA synthesis rate can be used to evaluate cell growth ability at the molecular level. [^3^H] thymidine deoxyribose ([^3^H]‐TdR), 5‐bromo‐2‐deoxyuridine (BrdU), and 5‐ethynyl‐2'‐deoxyuridine (EdU) are the most frequently used methods.^[^ ^94^, ^95^ ^]^ Radioactive [^3^H]‐TdR can be added to the culture medium as a raw material for DNA synthesis, which is absorbed into the transformed cells. Here, the response of cells to metal‐based NPs can be objectively characterized according to intracellular radioactive particles.^[^ ^96^ ^]^ For the BrdU and EdU methods, thymidine selectively binds to cell DNA in the S phase, which enables the detection of DNA synthesis.^[^ ^97^ ^]^

#### 3.2.2 Evaluation Methods for Metabolic Activity

Oxidative damage to cells can be quantitatively evaluated by detecting changes in metabolic activity or biosynthetic functions. The tetrazole‐reduction‐based strategy is the most commonly used method and includes MTT, XTT, water‐soluble tetrazolium salt‐1 (WST‐1), and cell counting kit‐8 (CCK‐8). The MTT assay, which is commonly used to assess cell activity, is based on the principle that living cells can selectively reduce tetrazolium salts to blue‐black formazan crystals, and the measured optical density (OD) can be directly related to the number of living cells.^[^ ^89^, ^98^, ^99^, ^100^ ^]^

**Figure 4:**
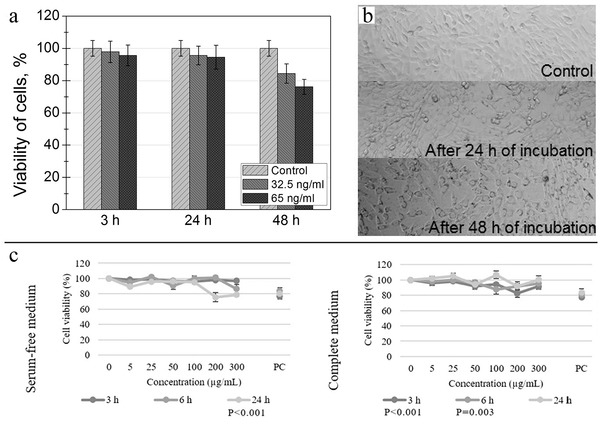
a) Cell viability comparison and b) corresponding XTT images of mouse embryonic fibroblasts NIH3T3 after incubating with 32.5 ng mL^‐1^ of SPIONs for different durations. Reproduced with permission.^[^ ^102^ ^]^ Copyright 2016, Molecular Diversity Preservation International (MDPI). c) Cell viability of the HepG2 cell line after exposure to TiO_2_ NPs assessed by the AB viability assay in serum‐free and complete media. Reproduced with permission.^[^ ^104^ ^]^ Copyright 2016, Elsevier.

XTT, WST‐1, and CCK‐8 are newly synthesized tetrazolium derivatives that are degraded by mitochondrial dehydrogenase in living cells to produce brown water‐soluble formazan. The OD of formazan is proportional to the number of living cells.^[^ ^101^ ^]^ Hence, cell proliferation can be assessed by directly measuring the spectral absorption OD. Greta et al. successfully analyzed the toxicity of superparamagnetic iron oxide NPs (SPIONs) based on XTT by measuring the activity of mitochondrial enzymes in viable cells (**Figure** **4**a,b).^[^ ^102^ ^]^ Remzova et al. found that cytotoxicity estimated using WST‐1 (cell metabolism) differed from that of LDH (cell integrity), with the cytotoxicity curve measured by LDH being far less distinct than that by WST‐1. The authors noted that this discrepancy may have resulted from the limited time in which LDH could be released into the cell culture medium.^[^ ^103^ ^]^

The AB assay uses a single reagent that can continuously and rapidly detect the metabolic activity of cells. Live cell mitochondrial enzymes can convert the blue oxidation form of AB into the red reduction form, whereas inactive cells cannot reduce AB; thus, one can evaluate metabolic activity by quantifying changes in fluorescence.^[^ ^105^ ^]^ Accordingly, the AB method is commonly used as an assay of cellular metabolic activity to evaluate the cytotoxicity of iron‐oxide‐based magnetic NPs.^[^ ^106^ ^]^ In low cell concentrations, the AB assay usually exhibits higher sensitivity than the MTT assay.^[^ ^107^ ^]^ In addition, because of the high adsorption or scattering properties of NPs, some metal oxide NPs may interact with analytical components or dyes and interfere with optical analysis. Among the metal‐based NPs, NiO and TiO_2_ NPs have excellent anti‐interference characteristics and do not interact normally with AB.^[^ ^93^, ^104^ ^]^

#### 3.2.3 Evaluation Methods for Apoptosis

Apoptosis induced by metal‐based NPs inhibits cellular activity. In the evaluation of apoptosis, researchers generally employ annexin V because of its high calcium‐dependent affinity and selectivity to lipids.^[^ ^108^ ^]^ Annexin V is used to detect phosphatidylserine (PS), which is an integral part of the plasma membrane that is restricted to the inner lobule of healthy cells. When PS translocates to the outer lobe of the plasma membrane during apoptosis, it can be measured using fluorescently labeled annexin V conjugates. Another strategy to evaluate cell death is to detect the uptake of DNA‐embedded fluorescent agents, such as propidium iodide (PI). There are many methods for directly identifying dead cells by combining fluorescent and DNA‐embedding dyes. Among them, the annexin V‐FITC/PI double staining method is widely used to distinguish cells in the early stages of apoptosis from dead cells. Almutairi et al. used annexin V‐FITC/PI double staining to identify apoptotic cells after exposure to Zn NPs.^[^ ^109^ ^]^ Mahmoud et al. and Yin et al. also reported apoptosis induced by MgO NPs and Ag NPs by double staining.^[^ ^110^, ^111^ ^]^

Besides the annexin V‐and PI‐based detection methods, fluorescent dye probes combined with flow cytometry are commonly used to detect mitochondrial membrane potential (MMP), which is closely related to apoptosis. When the morphology of mitochondria does not change significantly, its membrane potential has changed.^[^ ^112^ ^]^ Fluorescent dye probes combined with flow cytometry are the commonly used approach to detect mitochondrial membrane potential (MMP). Lipophilic cationic fluorescent dyes are tools for evaluating MMPs induced by metal NPs and include Rhod123,^[^ ^113^, ^114^ ^]^ tetramethylrhodamine ethyl ester (TMRE),^[^ ^115^ ^]^ and JC‐1.^[^ ^116^, ^117^ ^]^

### 3.3 Cytotoxicity Mechanism‐Based Evaluation Method

The cytotoxicity mechanisms of metal‐based NPs are mainly associated with oxidative stress, inflammatory response, autophagy, and apoptosis. Oxidative stress is typically evaluated according to ROS and superoxide dismutase (SOD) levels, the inflammatory response is evaluated by inflammatory factors, and autophagy and apoptosis are detected by DNA damage (**Table** **3**).

**Table 3:** Cytotoxicity mechanism‐based detection methods and their features

Index	Method	Advantages	Limitations	Refs.
ROS	DCFH‐DA	Simple, sensitive	Cannot distinguish ROS	[156]
Inflammatory factors	ELISA	Rapid, sensitive, easy to standardize	Poor repeatability, easily disturbed	[67]
DNA damage	Comet	High sensitivity, low cost and sample requirement	Subjective, manual operation	[157]
	MN	High sensitivity, simple and versatile	Possibly false micronucleus and false‐positive results	[158]
	TUNEL	High specificity and sensitivity	Subjective, poor specificity	[159]

#### 3.3.1 Evaluation Methods for Oxidative Stress

Living cells constantly adjust their gene expression patterns in response to changing internal and external conditions. A simple method for detecting oxidative stress or stimuli is to transfer signal molecules from one steady‐state level to another.^[^ ^126^ ^]^ Fluorescent probe 2',7'‐dichlorodihydrofluorescein diacetate (DCFH‐DA) is commonly used to detect intracellular ROS levels as DCF fluorescence can be monitored in intact cells.^[^ ^127^ ^]^ DCFH‐DA itself has no fluorescence and can freely pass through the cell membrane. After entering the cell, it is hydrolyzed by intracellular esterases to generate DCFH, which cannot penetrate the cell membrane. Intracellular ROS oxidizes non‐fluorescent DCFH to produce fluorescent DCF. Hence, oxidative stress can be evaluated by detecting DCF fluorescence yields or intracellular ROS levels.^[^ ^128^ ^]^ The DCFH‐DA assay is widely used to detect ROS induced by metal‐based NPs.^[^ ^129^, ^130^, ^131^ ^]^

#### 3.3.2 Evaluation Methods for Inflammatory Factors

**Figure 5:**
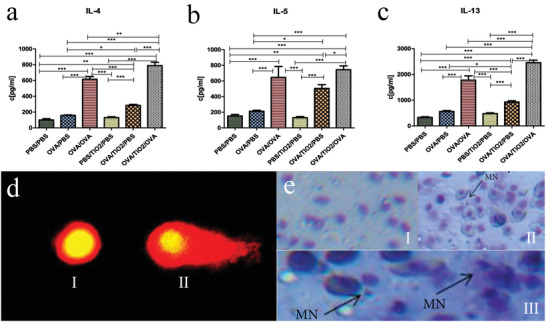
a–c) The Th2 (IL‐4, IL‐5, and IL‐13) cytokines results revealed the significant differences induced by TiO_2_ NPs in cytokine levels among the research groups. Reproduced with permission.^[^ ^136^ ^]^ Copyright 2020, Springer Nature. d) Comet pictures illustrating the effects of TiO_2_ NPs (150 mg kg^‐1^ body weight) on the extent of DNA damage: I) Control group, II) NPs group. Reproduced with permission.^[^ ^138^ ^]^ Copyright 2021, Springer Nature. e) The mutagenic effect of CdO NPs on fish: I) Control group. II) MN in erythrocytes of *Oreochromis mossambicus* at sublethal II (1/4 of LC50–10 µg mL^‐1^) for 21 d. III)MN in erythrocytes of *O. mossambicus* at sublethal III (1/2 of LC50–20 µg mL^‐1^) for 21 d. Reproduced with permission.^[^ ^139^ ^]^ Copyright 2020, Hindawi.

The inflammatory response induced by metal‐based NPs involves a variety of cytokines (e.g., TNF‐*α*, IL‐1*β*, and IL‐6) that can be detected using enzyme‐linked immunosorbent assay (ELISA). ELISA uses covalently bound enzyme–antibody molecules to detect and amplify antigen–antibody reactions, while the presence of enzymes is detected by adding an appropriate substrate, such as tetramethyl benzidine,^[^ ^132^ ^]^ *para*‐nitrophenyl phosphate,^[^ ^133^ ^]^ or *ortho*‐phenylenediamine.^[^ ^134^ ^]^ These detection systems are designed to produce color changes that can be quantified using a microtiter plate reader.^[^ ^135^ ^]^ ELISA was previously used to analyze the local effects of TiO_2_ NPs on ovalbumin (OVA)‐induced allergic airway inflammation in a mouse model. The control (phosphate‐buffered saline (PBS)/PBS, OVA/PBS, and OVA/OVA) and treatment groups (PBS/TiO_2_/PBS, OVA/TiO_2_/PBS, and OVA/TiO_2_/OVA) consisted of 5 and 10 mice, respectively. The results showed that TiO_2_ NPs induced a significant increase in Th2 cytokines, including IL‐4, IL‐5, and IL‐13 (**Figure** **5**a–c).^[^ ^136^ ^]^ ELISA can also be used to evaluate the influence of ultrasmall SPIONs on liver function.^[^ ^137^ ^]^

#### 3.3.3 Evaluation Methods for DNA Damage

The integrity of cell DNA is affected by various factors, such as ultraviolet rays, ionizing radiation, and alkylating agents. Metal‐based NPs can cause different levels of DNA damage.^[^ ^140^, ^141^ ^]^ Many approaches to evaluate DNA damage have been developed, including a comet assay, micronucleus assay, and terminal deoxynucleotidyl transferase deoxyuridine triphosphate (dUTP) nick end labeling (TUNEL) assay. The comet assay can accurately assess DNA repair function and detect teratogenic substances over time. It should be noted that the DNA damaging reagents used in this study can be removed from the cells, but the NPs cannot be easily removed. Because of its simplicity and sensitivity, the comet assay is widely used in many frontier fields, including biological monitoring,^[^ ^142^, ^143^ ^]^ DNA repair,^[^ ^144^, ^145^ ^]^ genotoxicity evaluation,^[^ ^146^, ^147^ ^]^ and extensive studies on the cytotoxicity of metal‐based NPs (Figure 5d).^[^ ^138^ ^]^

The micronucleus (MN) assay is another genotoxicity evaluation method used to detect chromosomal or mitotic organ damage (Figure 5e). In contrast to the comet assay, which detects interphase and DNA damage in mitotic cells, the MN assay only detects genetic damage in mitotic cells.^[^ ^148^ ^]^ The MN assay has some notable disadvantages. For example, the formation of the cell cytoskeleton and endocytosis of NPs can be affected by the addition of cytochalasin B to block cytokinesis, which interferes with the experimental results.^[^ ^149^ ^]^ Given that this is one of the most reliable, well‐established, and feasible genotoxicity tests, many studies on metal‐based NPs employ this method.^[^ ^150^, ^151^, ^152^ ^]^

The principle of the TUNEL method is that apoptotic cell DNA is cleaved by activated endonucleases to produce DNA gaps, thereby exposing a large number of 3'‐OH ends. Terminal deoxyribonucleotidyl transferase attaches the labeled dUTP to the 3'‐OH, and apoptosis is evaluated using flow cytometry and fluorescence microscopy.^[^ ^153^ ^]^ Hence, the TUNEL assay method can be used to evaluate DNA damage induced by various metal NPs.^[^ ^154^, ^155^ ^]^

## 4 Cytotoxicity of Metal‐Based NPs

Metal‐based NPs have unique physical and chemical properties that cause different biochemical activities, and exhibit different toxicities from those of ordinary metal materials (e.g., Li and Na).^[^ ^160^, ^161^ ^]^ The size of a metal‐based NP is comparable to that of protein molecules; thus, they can easily penetrate cell membranes and interact with protein molecules or subcellular organelle structures, ultimately causing neurotoxicity, immunotoxicity, and genotoxicity. With the extensive application of nanotechnology and NPs, the biosafety of these new materials and technologies has attracted much attention. To elucidate the potential risks, we systematically summarize the typical toxicity characteristics of different metal‐based NPs at the tissue and cellular levels.

### 4.1 Neurotoxicity

The central nervous system (CNS) is one of the target organs of metal‐based NPs, with the hippocampus being its most sensitive part.^[^ ^162^, ^163^, ^164^ ^]^ Metal‐based NPs induce the release of ROS and cytokines into the nervous system. These elements can damage the blood‐brain barrier (BBB) and cause CNS dysfunction (**Table** **4**).^[^ ^165^, ^166^ ^]^

**Table 4:** Typical neurotoxicity induced by different metal‐based NPs

Materials	Dose [mg L^‐1^]	Model	Typical neurotoxic effects	Refs.
Al_2_O_3_	6.25–100	Zebrafish	Impaired learning and memory	[183]
Ag	5, 10, 50	*Lymnaea*	Memory formation was blocked	[184]
TiO_2_	0.01, 0.1, 1.0	Zebrafish	Inhibition of neurodevelopment and motor neuron axonal growth	[185]
TiO_2_	0.1, 1.0	Zebrafish	Neuronal damage	[186]
TiO_2_	0.1	Zebrafish	Aggravated abnormity of swimming behavior and social behavior	[166]
Ag	0.02	BBB model	Damaged endothelial cells	[187]

#### 4.1.1 Routes into the Central Nervous System

The interface between the cardiovascular and nervous system is characterized by a unique barrier, the BBB. The main function of this barrier is to maintain brain homeostasis and regulate the inflow and outflow transport to protect against injury.^[^ ^167^ ^]^ Only small or low‐molecular‐weight substances can cross the BBB and enter the CNS through passive diffusion, active transport, or endocytosis. Owing to their small size and unique physical and chemical properties, metal‐based NPs can easily pass through the BBB and affect the neural reflex activity of the cerebral cortex.^[^ ^168^ ^]^ Grissa et al. evaluated the neurotoxicity of orally administered TiO_2_ NPs in rats and found that the NPs accessed the brain through the BBB. Accumulation of TiO_2_ NPs in the brain caused oxidative stress, histopathological damage, and increased the NO levels. In addition, the toxic effect increased in a dose‐dependent manner.^[^ ^169^ ^]^ Dhakshinamoorthy et al. assessed the damage caused by Fe_2_O_3_ NPs on BBB permeability and their accumulation in the brain using Evans blue staining, iron estimation, and Prussian blue staining.^[^ ^72^ ^]^

**Figure 6:**
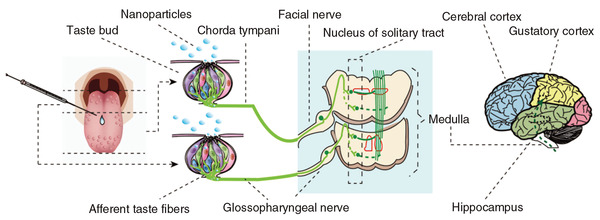
Pathway for the translocation of NPs (ZnO and TiO_2_) to the brain by tongue instillation. Reproduced with permission.^[^ ^172^ ^]^ Copyright 2017, Future Medicine Ltd.

Metal‐based NPs can also enter the CNS along the olfactory nerve to be distributed in different brain regions (**Figure** **6**). Through intranasal infusion of Al_2_O_3_ NPs in rats, a previous study showed that the Al_2_O_3_ NPs were transferred to the brain through the olfactory nerve pathway and then accumulated in the hippocampus, olfactory bulb, cerebral cortex, and striatum where they caused ultrastructural changes, oxidative damage, inflammation, and histopathological damage.^[^ ^170^ ^]^ This route was confirmed by ZnO NP delivery experiments.^[^ ^171^ ^]^

#### 4.1.2 Neurotoxic Effects

The neurotoxicity induced by metal‐based NPs is mainly reflected at the neurobehavioral, histopathological, and neurotransmitter levels. Neurobehavioral changes are commonly used in the evaluation of metal‐based NP‐induced neurotoxicity. For instance, by analyzing the social behavior as well as the learning, memory, and motor coordination abilities of mice injected with Ag NPs, Greish found that exposure to Ag NPs not only reduced their social interaction and exploration activities, but also impaired their memory, learning, and motor functions.^[^ ^173^ ^]^ Tian et al. showed that ZnO NPs could cause cognitive impairment and pathological changes in the hippocampus (a brain region closely associated with learning and memory). In particular, cognitive impairment was greater in old‐adult mice than that in adult mice.^[^ ^174^ ^]^ SPIONs, which are widely used as theranostic drug carriers and MRI contrast agents, may also have neurotoxic effects on the dorsal striatum and hippocampus.^[^ ^175^ ^]^

**Figure 7:**
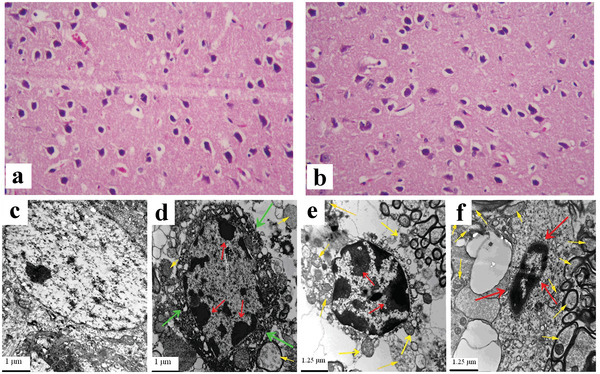
Histological images of mice brain of a) the control group and tissue after treating with b) ZnO NPs. Reproduced with permission.^[^ ^176^ ^]^ Copyright 2020, Elsevier. c–f) Ultrastructure of hippocampal cell of mice caused by nasal administration of TiO_2_ NPs for 90 consecutive days. c) Control group. d) 2.5 mg kg^‐1^ body weight TiO_2_ NPs. e) 5 mg kg^‐1^ body weight TiO_2_ NPs. f) 10 mg kg^‐1^ body weight TiO_2_ NPs. Green arrows indicate irregularity of nuclear membrane, significant shrinkage of the nucleus. Red arrows suggest chromatin marginalization. Yellow arrows exhibit mitochondria swelling. Reproduced with permission.^[^ ^177^ ^]^ Copyright 2014, Wiley‐VCH.

Histopathology can intuitively explain organic pathological changes caused by endogenous or exogenous factors. In studies of neurotoxic effects related to metal‐based NPs, the observation and analysis of histopathological effects are often conducted. Using this approach, Dkhil et al. found that ZnO NPs induced perineural vascularization and vascular congestion (**Figure** **7**a,b).^[^ ^176^ ^]^ TiO_2_ NPs could also be translocated and accumulated in the brain, leading to oxidative stress, over‐proliferation of glial cells, tissue necrosis, and hippocampal cell apoptosis (Figure 7c–f).^[^ ^177^ ^]^

Neurotransmitters are specific chemicals that can transmit information between the synapses of neurons, and metal‐based NPs can alter neurotransmitter secretion levels. For example, when *γ*‐aminobutyric acid (GABA, an inhibitory neurotransmitter) binds to related receptors, the complexes can induce an influx of chloride ions that results in neuronal hyperpolarization and inhibition of nerve signal transduction. Guan et al. found that TiO_2_ NPs caused intense neurotoxicity in the blood of clams by increasing the in vivo neurotransmitter GABA concentration.^[^ ^178^ ^]^

The neurotoxic effects of metal‐based NPs are not limited to adults. Studies have found that metal‐based NPs also have a potential neurotoxicity risk during infancy and puberty.^[^ ^179^, ^180^ ^]^ The offspring of mothers exposed to metal‐based NPs are also at risk of neurotoxic effects.^[^ ^181^, ^182^ ^]^

### 4.2 Immunotoxicity

The immune system is sensitive to foreign substances and can quickly respond to and resist any harmful effects. According to the difference in reaction speed and specificity, immunity involves innate and adaptive responses with many interactions.^[^ ^188^ ^]^ Compared to normal materials, metal‐based NPs are more likely to enter organisms and interact with each other. Once inside an organism, the physical and chemical properties of metal‐based NPs would determine not only their effect but also the manner in which the immune system removes these exogenous substances. **Table** **5** summarizes examples of metal‐based NP‐induced immunotoxicity.

**Table 5:** Immunotoxicity induced by typical metal‐based NPs

Materials	Dose [mg kg^‐1^]	Model	Immunotoxicity	Refs.
ZnO	350	Rats	Considerable degree of immunotoxicity	[205]
Cu	100, 200, 400	Rats	Liver fibrosis immunosuppressive effects	[206]
Cu	50, 100, 200	Rats	Suppresses the immune function of the spleen	[20]
TiO_2_	300, 1200	Mice	Th1/Th2 imbalance	[207]
TiO_2_	1.25, 2.5, 5	Mice	NF‐*κ*B‐mediated immunotoxicity	[202]
Ag	2.0, 6.0	Rats	Affects multiple immune parameters	[208]

#### 4.2.1 Toxicity to Innate Immunity

**Figure 8:**
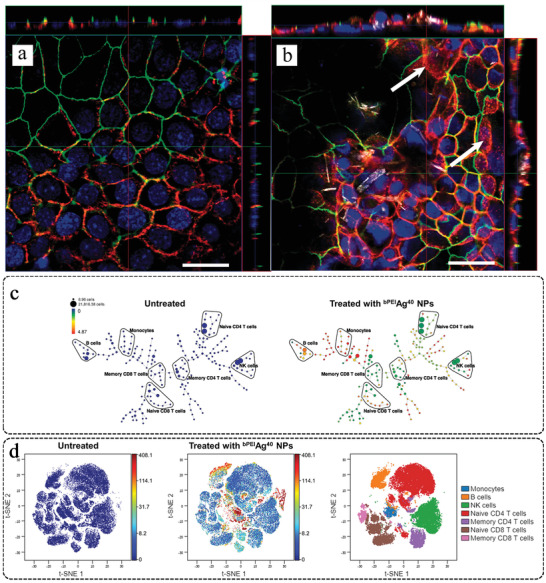
Optical images of a) untreated and b) TiO_2_ NP‐treated (80 µg cm^‐2^) 16HBE cell monolayers. TiO_2_ exposure can cause a marked and widespread perturbation of the distribution of claudin‐7 (red in the online version) and ZO‐1 (green in the online version). Reproduced with permission.^[^ ^191^ ^]^ Copyright 2020, Taylor & Francis Group. c) SPADE (spanning‐tree progression analysis of density‐normalized events) trees for control and Ag NP‐treated samples. The SPADE trees show that the cell‐associated Ag NPs were not evenly distributed for all cell types. d) The t‐SNE (t‐distributed stochastic neighbor embedding) results support the findings in SPADE trees, namely, cellular Ag NP association is high in monocytes and B cells, but medium in T cells and NK cells. In t‐SNE plots, the color gradient representing the cell‐associated Ag amount and positions of each cell type are displayed in the right‐most figure. Reproduced with permission.^[^ ^193^ ^]^ Copyright 2020, Wiley‐VCH.

Innate immunity is the first line of defense against pathogens and is essential for maintaining homeostasis, preventing microbial invasion, eliminating multiple pathogens, and promoting adaptive immune responses. The immune components include physical and chemical barriers, body fluids, and cell‐mediated components.^[^ ^189^ ^]^ The respiratory mucosa, a mechanical barrier, is the first line of defense in the respiratory system. Under normal circumstances, once the airway surface mucus inhales small exogenous substances, cilia would discharge them through the regular swing. However, long‐term exposure to metal‐based NPs can alter the functionality of the mucociliary system, and weaken its ability to remove exogenous particles. Ultimately, mucosal edema, epithelial cell proliferation, vascular expansion, and inflammatory cell secretion occur in the respiratory system. Cho et al. investigated the toxicity of ZnO NPs to the innate immune system by injecting ZnO NPs into the lungs of rats via the trachea. They found that ZnO NPs could induce eosinophilia, airway epithelial cell proliferation, goblet cell proliferation, and pulmonary fibrosis.^[^ ^190^ ^]^ TiO_2_ NPs could cause immunotoxicity in bronchial 16HBE cells by increasing the permeability of the 16HBE monolayer and disturbing the distribution of the tight junction protein ZO‐1 (**Figure** **8**a,b).^[^ ^191^ ^]^ Even with brief exposure to ZnO NPs, the frequency and intensity of airway irritation symptoms (throat irritation and cough) increased in healthy volunteer subjects and the levels of neutrophils and cytokines (i.e., IL‐6 and IL‐8) increased in sputum samples.^[^ ^192^ ^]^

Alveolar macrophages (AMs), natural killer (NK) cells, and dendritic cells (DCs) are the innate immune cells. AMs are the first line of defense against foreign substances that invade the lungs. They participate in innate immune regulation and play an important role in homeostasis during pulmonary infection, lung cancer, and chronic inflammatory disease.^[^ ^194^ ^]^ CeO_2_ NPs have been reported to increase the response of AMs to LPS attack and induce apoptosis of AMs by activating caspase‐9 and ‐3.^[^ ^195^ ^]^ NK cells are innate immune system lymphocytes known for their ability to mediate cytotoxicity and produce cytokines after connecting the activation receptors encoded by the reproductive system.^[^ ^196^ ^]^ After long‐term exposure to TiO_2_ NPs, the number of NK cells decreased significantly in a mouse model.^[^ ^197^ ^]^ Finally, DCs are innate immune cells that recognize pathogen‐ and danger‐related signals to form an acute inflammatory response. DCs connected the innate and adaptive immune systems.^[^ ^198^ ^]^ This function of DC is affected by TiO_2_ NPs, which can induce the upregulation of MHC‐II, CD80, and CD86 in DCs.^[^ ^199^ ^]^ These results prove that metal‐based NPs may weaken the immune defense function of the body by affecting the functions of AMs, NK cells, and DCs.

#### 4.2.2 Toxicity to Adaptive Immunity

The immune system constitutes innate and adaptive immunity, which collaborates to maintain the host defense against pathogens.^[^ ^200^ ^]^ B lymphocytes are the main components of humoral immune function. They produce antibodies and cytokines that present antigens to T lymphocytes and regulate the immune response.^[^ ^201^ ^]^ Several reports have indicated that metal‐based NPs can decrease B lymphocyte counts.^[^ ^20^, ^197^, ^202^ ^]^ T lymphocytes are the main cells involved in adaptive immune response. They coordinate multiple aspects of adaptive immunity, including responses to pathogens, allergens, and tumors.^[^ ^203^ ^]^ However, metal‐based NPs can affect the function of the immune system by altering the proliferation and phenotype of T lymphocytes. For example, long‐term ingestion of TiO_2_ NPs aggravated dextran sulfate sodium salt‐induced chronic colitis and immune response. Here, the accumulation of TiO_2_ NPs reduced the number of CD4^+^ T cells and regulated T cells and macrophages in mesenteric lymph nodes.^[^ ^204^ ^]^ In addition, the elemental detection capability of mass cytometry enabled direct quantitative measurement of cellular Ag NP associations, as monocytes and B cells have an especially high affinity for ^bPEI^Ag^40^ NPs (Figure 8c,d). B cells and naive CD4^+^ T cells displayed different levels of cellular responses and viability loss with respect to their cellular Ag NP dose.^[^ ^193^ ^]^

### 4.3 Genotoxicity

Genotoxicity describes substances that have destructive effects on genetic material (i.e., DNA and RNA) and affect cell integrity. The study of genotoxicity is an important part of cancer research and risk assessment of potential carcinogens. Genotoxicity is often confused with mutagenicity. In fact, all mutagens are genotoxic, but not all genotoxic substances are mutagenic.^[^ ^209^ ^]^ Ascribing to their nanoscale size, metal‐based NPs can enter the nucleus and directly interfere with the structure and function of genomic DNA, ultimately resulting in genotoxicity. However, the complete mechanism of this genotoxic effect has not been determined yet. At present, research is focused on the direct effect of NPs on genetic materials, the associated generation of ROS, and the resulting DNA and chromosome damage.^[^ ^210^, ^211^, ^212^ ^]^ Here, we summarize the genotoxicity induced by metal‐based NPs at the DNA and chromosomal levels (**Table** **6**).

**Table 6:** Typical genotoxicity induced by different metal‐based NPs

Type	Dose [mg L^‐1^]	Model	Genotoxicity	Refs.
Ag	0.0125, 0.125	*Saccostrea glomerata*	DNA strand break	[141]
Ag	1.0–25	Mononuclear cells	Genotoxic potential	[218]
Ag	0.004–0.024	*Lymnaea*	Dose‐dependent DNA damage	[219]
ZnO	10–200	Osteoblasts	Induce DNA damage	[220]
ZnO	10–120	Zebrafish embryos	Oxidative DNA damage	[221]
TiO_2_	2.56	Mice	Blood DNA damage	[8]

**Figure 9:**
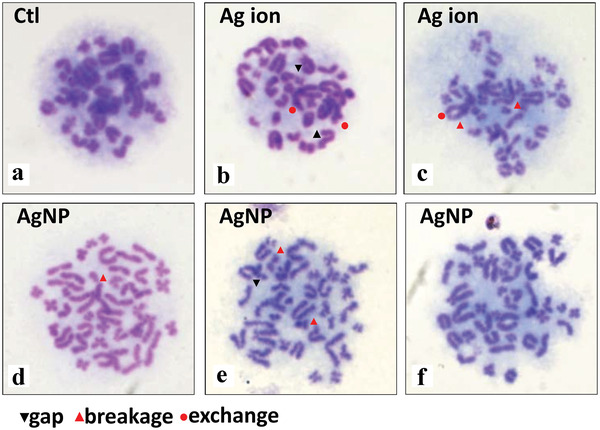
Chromosome aberrations induced by Ag NPs and Ag ions in rat bone marrow cells. a) Vehicle control. Structure aberrations include gap, breakage, and exchange can be observed in both (b,c) Ag ion and Ag NPs treated groups (d–f) as indicated in each panel, and a phenomenon with increase of chromosome count in single cell was observed in the latter. Reproduced with permission.^[^ ^216^ ^]^ Copyright 2017, PLOS.

In genotoxicity studies of metal‐based NPs, comet assay and micronucleus test are the frequently used toxicological methods. Using the comet and MN assays, Al‐Abdan et al. investigated the genotoxicity of CdO NPs in aquatic organisms and found that DNA fragments and loose DNA loops migrated toward the anode and formed a comet's tail.^[^ ^139^ ^]^ Hou et al. studied genotoxicity in zebrafish and found that ZnO NPs inhibited the expression of cyclin (Cyc), cyclin‐dependent kinase (CDK), and microchromosome maintenance (MCM); activated the Cyc/CDK complex (CycD/CDK4,6, CycE/CDK2, CycA/CDK2); caused MCM to fail at different stages (G1, M, and G2); and ultimately led to DNA replication disorder.^[^ ^213^ ^]^ The most serious type of DNA damage is DNA double‐strand break, which, if the repair process is ineffective, can cause cell death, chromosome translocation, or loss of genetic information. Studies have shown that human amniotic epithelial cells exposed to TiO_2_ NPs can undergo DNA double‐strand breaks.^[^ ^214^ ^]^ To investigate the influence of TiO_2_ NPs on chromosomes, Ali et al. fed mice different doses (50, 250, and 500 mg kg^‐1^ body weight) of TiO_2_ NPs (21 and 80 nm) for five consecutive days. They found that the chromosomes generated aberrations at a rate that was positively correlated with the feeding dose. In addition, TiO_2_ NP_S_ with a particle size of 21 nm showed a higher frequency of genetic aberrations and worse histological results.^[^ ^215^ ^]^ Ag NPs and Ag^+^ could cause chromosomal damage to bone marrow cells, mainly in the form of chromosome or chromatid breakages (**Figure** **9**).^[^ ^216^ ^]^

Histone H2AX phosphorylation (*γ*‐H2AX) is considered a suitable indicator of DNA damage, especially DNA double‐strand breaks. Using *γ*‐H2AX, Choi et al. confirmed that Ag NP exposure can induce DNA damage in adult zebrafish liver cells.^[^ ^217^ ^]^

### 4.4 Reproductive Toxicity

Reproductive toxicity occurs when chemicals adversely affect the normal function and development or reproductive behavior of male or female reproductive tissues, resulting in decreased fertility.^[^ ^222^ ^]^ To date, a large number of animal experiments have shown that exposure to metal‐based NPs adversely affects the reproductive system. The reproductive toxicity of metal‐based NPs in mammals is mainly reflected in genital damage, inhibition of sex hormones, and their impact on germ cells and future generations (**Table** **7**).

**Table 7:** Typical reproductive toxicity induced by different metal‐based NPs

Materials	Dose [mg kg^‐1^]	Model	Typical reproductive toxicity	Refs.
Au	0.5	Mice	Decrease in testosterone production, downregulation of 17*α*‐hydroxylase	[234]
Ag	1.0	Mice	Affects various gene expressions of fetal ovaries	[235]
CeO_2_	20, 40	Mice	Reduced testis weight and sperm production and motility	[230]
Ni	5, 15, 45	Rats	Significant reproductive toxicity	[223]
TiO_2_	1.25, 2.5, 5	Mice	Inhibits follicle development	[236]
TiO_2_	2.5, 5, 10	Mice	Premature ovarian failure	[232]

#### 4.4.1 Toxicity to Male Reproduction

**Figure 10:**
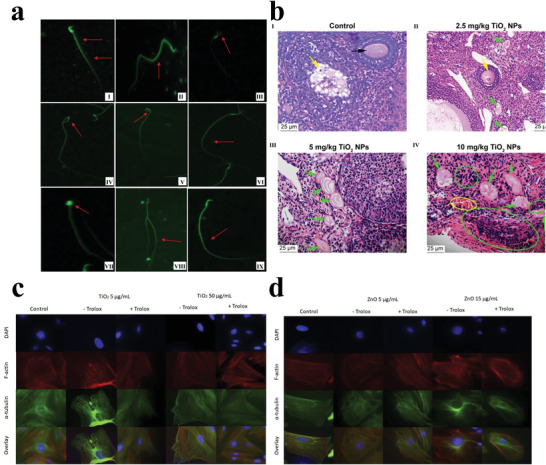
a) Morphological changes of spermatozoa induced by NiO NPs. (I) normal morphology. (II–IX) Perms of NiO NP‐treated rats show an amorphous, knobbed, hooked, or detached head. while some of the tails of few sperms exhibit a spiral, bifurcated or broken tail. Reproduced with permission.^[^ ^226^ ^]^ Copyright 2021, Springer Nature. b) Histopathological examination of mice ovary following gavage administration of TiO_2_ NPs for 30 d. (I) unexposed mice present normal development of primary follicles (black arrow) and secondary follicles (yellow arrow). (II) 2.5 mg kg^‐1^ TiO_2_ NPs exposed group shows atrophic secondary follicle (yellow arrow), primary follicle atresia (green arrow), and apoptosis of granule cells (blue arrow). (III) 5 mg kg^‐1^ TiO_2_ NPs exposed group shows large primary follicle atresia (green arrow) and granule cell apoptosis (blue arrow). (IV) 10 mg kg^‐1^ TiO_2_ NPs exposed group shows severe inflammatory cell infiltration (green circle), congestion (yellow circle), significant primary follicle atresia (green arrow) and disposed disorder or apoptosis of granule cells (blue arrow). Reproduced with permission.^[^ ^232^ ^]^ Copyright 2018, National Center for Biotechnology Information, U.S. National Library of Medicine. c,d) Representative micrographs of TiO_2_ and ZnO NP effects on cytoskeletal organization in theca cells after culture with or without Trolox. Reproduced with permission.^[^ ^233^ ^]^ Copyright 2020, Elsevier.

Researchers have found that gavage, intravenous injection, and subcutaneous or intratesticular injection of metal‐based NPs resulted in distinct toxic effects on the reproductive system of male animals.^[^ ^223^, ^224^, ^225^ ^]^ These reproductive toxicity effects include an increase in the sperm deformity rate and a decrease in testicular weight, sperm concentration, testicular histopathology, testosterone levels, and marker enzyme activity. For example, NiO NPs are widely used in electronic devices owing to their versatility; however, they can cause reproductive toxicity in male rats, resulting in moderate to severe toxicity in the testis, epididymis, vas deferens, seminal vesicles, and prostate (**Figure** **10**a).^[^ ^226^, ^227^ ^]^ In addition, the mRNA expression of some steroidogenic genes, such as Star, P450scc, P450c17, 3*β*‐Hsd, and 17*β*‐Hsd were down‐regulated.^[^ ^228^, ^229^, ^230^ ^]^ For example, following tube feeding with ZnO NPs for 3 d, the reproductive organs of mice exhibited obvious accumulation of zinc. The accumulated ZnO NPs not only induced a decrease in testicular weight, but also caused an imbalance in the hematological and serum biochemical parameters of male mice. Furthermore, histopathological examination revealed structural disorders, apoptosis, and cell death. In the ovaries, ZnO NPs induced cell apoptosis in the Shh pathway‐activated ovary cells and affected steroidogenesis. A comparative study of ovarian and testicular damage confirmed that the reproductive toxicity of ZnO NPs was more severe in male mice than that in female mice.^[^ ^231^ ^]^

#### 4.4.2 Toxicity to Female Reproduction

Metal‐based NP exposure adversely affects the reproductive system of female animals. Hong et al. continuously exposed female mice to 2.5, 5, or 10 mg kg^‐1^ TiO_2_ NPs via tube feeding. After 30 d, the female mice exhibited premature ovarian failure characterized by a reduction in reproductive ability along with decreased levels of inhibin B, estradiol, and progesterone, among others (Figure 10b).^[^ ^232^ ^]^ In a comparative study of the effects of TiO_2_ and ZnO NPs on ovarian antral follicle growth and ultrastructure, TiO_2_ NPs were internalized and aggregated in the cells, which increased hair follicle diameter and disrupted cytoskeletal alignment, but was partially blocked by Trolox. However, ZnO NPs, which were partially dissolved in the culture medium, reduced follicle diameter and disrupted cytoskeleton arrangement, without being blocked by Trolox (Figure 10c,d).^[^ ^233^ ^]^ In addition, Kuang et al. found that ZnO NPs could activate mitochondria‐mediated signaling pathways and induce caspase‐dependent damage to the uterus.^[^ ^231^ ^]^ Hence, the ovarian follicular toxicity induced by different metal‐based NPs may be due to different toxicity mechanisms.^[^ ^233^ ^]^

### 4.5 Other Toxicity Effects

Metal‐based NPs have become key components in various areas of research and industry, including agriculture, biomedicine, and the production of personal consumer goods.^[^ ^237^, ^238^, ^239^ ^]^ However, their vast application increases the exposure risk and potential cytotoxicity of not only the above four systems but also of other organs and tissues, including the lungs, kidneys, heart, and skin. For instance, the synergistic effect of hypertension and inhalation of NPs can lead to irreversible hemodynamic damage associated with cardiac structural damage and may ultimately cause heart failure after six weeks of TiO_2_ NP exposure.^[^ ^240^ ^]^ On the other hand, TiO_2_ NPs can also produce excessive ROS and further induce oxidative stress in human immortal skin keratinocyte cells and mouse skin.^[^ ^241^ ^]^ In ZnO NP exposure experiments, researchers found that NPs significantly increased DNA and chromosome damage in renal cells, which was accompanied by a decrease in catalase and glutathione S‐transferase activities. Cytotoxicity is also dependent on concentration.^[^ ^242^ ^]^ In addition, co‐exposure to multiple ambient NPs can affect their biodynamics. Under the simultaneous exposure of Au and Ag NPs, the lungs of rats exhibited both fast and slow phases in the clearance of Ag NPs, whereas the clearance of Au NPs was always associated with a slow phase. In this case, the clearance of Ag NPs was altered by the presence of Au NPs, perhaps due to some interaction between Ag and Au NPs affecting the dissolution and/or mechanical clearance of Ag NPs in vivo.^[^ ^243^ ^]^

## 5 Conclusion, Challenges, and Perspectives

In this review, we summarized the most recent and relevant studies in cytotoxicity induced by various metal‐based NPs. This paper should serve as a useful and accessible reference to scientists in this and related fields of study. The complex and diversified patterns of migration and transformation as well as continuous accumulation of metal‐based NPs can cause various toxic effects, and pose serious health risks to humans. Our understanding of the cytotoxicity induced by metal‐based NPs remains insufficient to describe their complete underlying mechanisms. Significant experimental and theoretical efforts have been directed toward understanding the toxicity effects of metal‐based NPs, but additional efforts are needed before they can be used in clinical medicine. Below, we propose potential directions and challenges to be addressed in future studies.

### 5.1 Interrelation between Cytotoxicity and Physical and Chemical Properties of Metal‐Based NPs

Many studies have shown that cytotoxicity induced by metal‐based NPs is related to particle size, specific surface area, crystal conformation, exposure mode, and chemical components, among others. Hence, to conclusively explain the cytotoxic effects of metal‐based NPs, comprehensive and systematic experiments are required. In addition, the parameters of metal‐based NPs must be controlled individually to validate the impact of each factor. The establishment of a structure–activity relationship model between the properties of metal‐based NPs and substances in the body can provide a scientific basis for comprehensive and accurate evaluation of the cytotoxicity of metal‐based NPs. For example, high‐throughput screening technology can effectively estimate the relationships between diverse physical and chemical parameters and cytotoxicity.^[^ ^244^ ^]^ Additionally, structure‐based computational molecular modeling is a promising tool for understanding and predicting the interaction between NPs and nanobiological systems.^[^ ^245^ ^]^

### 5.2 Experimental Conditions

The cytotoxicity induced by metal‐based NPs usually exhibits significant differences under different conditions, such as animal models, cell lines, concentration, exposure time, and temperature. Generally, high concentrations and prolonged exposure increase toxicity.^[^ ^246^, ^247^ ^]^ Some studies have reported that a decrease in pH can enhance the cytotoxicity of metal‐based NPs, and this mechanism may reduce pH values through the agglomeration of the NPs.^[^ ^248^, ^249^, ^250^ ^]^ Most modern studies have focused on short‐term effects. Long‐term (more than a few weeks or months) in vivo and in vitro experiments would be indispensable in establishing a more comprehensive description of oxidative stress, inflammation, and apoptosis under accumulating levels of metal‐based NPs. Co‐exposure to metal‐based NPs is another issue that has been largely overlooked. Given the extensive application of metal‐based NPs, humans are at particularly high risks of exposure. Hence, their interactions and synergistic effects may lead to unexpected cytotoxicity. Multiomics studies using proteomics, genomics, and metabolomics are promising strategies to assess the toxicity of metal‐based NPs at precise levels and should be used in future studies that consider more varied environmental conditions.

### 5.3 Experimental Models

Although healthy cells and animals are frequently used as experimental models of cytotoxicity, their use has some limitations in expounding the exposure risk and metabolic characteristics of metal‐based NPs. Since human patients can carry undiagnosed diseases and disorders, metal‐based NP exposure may exhibit different or even opposite cytotoxic effects to what research suggests. Hence, we should further reference the clinical data to confirm the experimental models before cytotoxicity experiments. A wider range of experimental models is necessary to verify the universality and specificity of the cytotoxicity induced by metal‐based NPs.

### 5.4 Modification of Metal‐Based NPs

Many publications reveal that there is a strong correlation between tissue penetration/accumulation of nanoparticles and their size: spheres less than 3 nm in diameter extravasate from tissues nonspecifically, those 3–8 nm in diameter undergo renal clearance, those 30–80 nm in diameter are more likely to be sequestered in the lungs (leaky vasculature), liver and spleen, and finally particles greater than 80 nm in diameter are generally trapped by the liver and spleen. In addition, some in vitro experiments have shown that small nanoparticles exhibit strong cytotoxicity.^[^ ^251^, ^252^, ^253^ ^]^ Particle shape is another factor that affects their cytotoxicity. Therefore, the appropriate size and shape modification of metal‐based NPs may mitigate cytotoxicity to a certain extent. In addition, surface chemical modification can serve as an effective approach to mitigate cytotoxicity by regulating the biological effects of metal‐based NPs.

## Conflict of Interest

The authors declare no conflict of interest.
